# Prevalence and clinical impact of malaria infections detected with a highly sensitive HRP2 rapid diagnostic test in Beninese pregnant women

**DOI:** 10.1186/s12936-020-03261-1

**Published:** 2020-05-24

**Authors:** Valérie Briand, Gilles Cottrell, Nicaise Tuike Ndam, Xavier Martiáñez-Vendrell, Bertin Vianou, Atika Mama, Bienvenue Kouwaye, Sandrine Houzé, Justine Bailly, Erasme Gbaguidi, Darius Sossou, Achille Massougbodji, Manfred Accrombessi, Alfredo Mayor, Xavier C. Ding, Nadine Fievet

**Affiliations:** 1 Institut de Recherche Pour le Développement (IRD), University of Bordeaux, Inserm, UMR 1219, 146 rue Léo-Saignat, 33076 Bordeaux Cedex, France; 2 Université de Paris, MERIT, IRD, 75006 Paris, France; 3 ISGlobal, Hospital Clínic of Barcelona, Universitat de Barcelona, Barcelona, 08036 Spain; 4 Institut de Recherche Clinique du Bénin (IRCB), Cotonou, Benin; 5 AP-HP, Centre National de Référence sur le paludisme, hôpital Bichat-Claude-Bernard, 75017 Paris, France; 6 Faculty of Infectious and Tropical Diseases, Disease Control Department, London School of Hygiene and Tropical Medicine, WC1E 7HT London, UK; 7 FIND, 1202 Geneva, Switzerland

**Keywords:** Malaria, Pregnancy, Africa, Diagnostic tests, HRP-2 antigen

## Abstract

**Background:**

While sub-microscopic malarial infections are frequent and potentially deleterious during pregnancy, routine molecular detection is still not feasible. This study aimed to assess the performance of a Histidine Rich Protein 2 (HRP2)-based ultrasensitive rapid diagnostic test (uRDT, Alere Malaria Ag Pf) for the detection of infections of low parasite density in pregnant women.

**Methods:**

This was a retrospective study based on samples collected in Benin from 2014 to 2017. A total of 942 whole blood samples collected in 327 women in the 1st and 3rd trimesters and at delivery were tested by uRDT, conventional RDT (cRDT, SD BIOLINE Malaria Ag Pf), microscopy, quantitative polymerase chain-reaction (qPCR) and Luminex-based suspension array technology targeting *P. falciparum* HRP2. The performance of each RDT was evaluated using qPCR as reference standard. The association between infections detected by uRDT, but not by cRDT, with poor maternal and birth outcomes was assessed using multivariate regression models.

**Results:**

The overall positivity rate detected by cRDT, uRDT, and qPCR was 11.6% (109/942), 16.2% (153/942) and 18.3% (172/942), respectively. Out of 172 qPCR-positive samples, 68 were uRDT-negative. uRDT had a significantly better sensitivity (60.5% [52.7–67.8]) than cRDT (44.2% [36.6–51.9]) and a marginally decreased specificity (93.6% [91.7–95.3] versus 95.7% [94.0–97.0]). The gain in sensitivity was particularly high (33%) and statistically significant in the 1st trimester. Only 28 (41%) out of the 68 samples which were qPCR-positive, but uRDT-negative had detectable but very low levels of HRP2 (191 ng/mL). Infections that were detected by uRDT but not by cRDT were associated with a 3.4-times (95%CI 1.29–9.19) increased risk of anaemia during pregnancy.

**Conclusions:**

This study demonstrates the higher performance of uRDT, as compared to cRDTs, to detect low parasite density *P. falciparum* infections during pregnancy, particularly in the 1st trimester. uRDT allowed the detection of infections associated with maternal anaemia.

## Background

In sub-Saharan Africa (SSA), around 39 million pregnant women are exposed to malaria every year [1]. Malaria in pregnancy (MiP) due to *Plasmodium falciparum* is one of the leading causes of maternal anaemia, low birthweight and fetal growth restriction, which are high risk factors for neonatal and infant morbidity and mortality [1, 2].

Several studies have evidenced high proportions of sub-microscopic infections—that are not detectable by microscopy because of low parasite densities—among pregnant women [3]. In Benin, this proportion was as high as 25% at the first antenatal care visit in early first-trimester [4]. Besides, these infections have been associated with a reduction in birth weight, as well as an increase in low birth weight and maternal anaemia [3, 5, 6], especially those occurring early in pregnancy or in primigravidae [3]. Although intermittent preventive treatment (IPTp) with sulfadoxine-pyrimethamine (SP) could theoretically clear and reduce the prevalence of such infections during pregnancy [3], resistance of parasites to SP and the low IPTp coverage in most SSA countries [1] are factors that limit its effectiveness. Besides, the administration of IPTp-SP is only recommended from the second trimester onwards. The accurate identification and treatment of women with sub-microscopic infections in the first trimester of pregnancy may be of high clinical relevance considering the high prevalence and significant deleterious effects of these early infections [7–9].

Sub-microscopic infections can be detected by nucleic acid amplification techniques (such as Polymerase Chain Reaction (PCR) or Loop-mediated isothermal Amplification), however these require highly trained staff, relatively sophisticated laboratory infrastructure and cannot be used in a point-of-care (POC) manner for malaria. Malaria rapid diagnostic test (RDT) are useful POC tool to screen pregnant women for malaria. Recently, a Histidine Rich Protein 2 (HRP2)-based ultrasensitive rapid diagnostic test (uRDT) has been made available (Alere Malaria Ag Pf ultra-sensitive RDT), with an analytical sensitivity (i.e. a detection threshold) ten times higher than conventional RDTs [10]. This test showed good performances compared to PCR in pregnant women in low transmission malaria areas [11, 12].

This study aimed to assess the performance of this uRDT, compared to conventional RDT (cRDT) and qPCR, for the detection of *P. falciparum* malaria in peripheral and placenta blood from pregnant women in Benin, a high malaria-endemic area. Also, the association of uRDT-positive/cRDT-negative infections with poor maternal and birth outcomes was assessed.

## Methods

### Study site and population

This retrospective study was performed using blood samples collected during the RECIPAL study conducted in Southern Benin (2014–2017) [13]. Briefly, RECIPAL aimed to assess the prevalence and consequences of malaria in the first trimester of pregnancy on maternal and child health. It was based on a cohort of 411 pregnant women who were recruited before conception and then followed monthly from early pregnancy to delivery. In April 2018, 378 out of the 411 pregnant women (92%) consented to have the samples collected during the RECIPAL study used for the present study. Among the remaining women, 20 had migrated to Nigeria, 9 refused to participate, 2 were lost to follow-up and 2 were deceased.

### Sample collection and handling

During the RECIPAL study, each month during pregnancy, EDTA preserved capillary blood samples were collected to detect malaria parasites using microscopy. At the same occasions, 50µL of whole blood was blotted on filter paper to make dried blood spot, which were dried, preserved at −20 °C and used for DNA extraction. The different types and volumes of blood that were used for microscopy, qPCR, RDTs and HRP2 level determination are presented in Additional file 1.

In addition, EDTA preserved venous blood samples were collected twice during pregnancy (at 1st and 3rd trimester, for haemoglobin determination), as well as twice at delivery (one sample from maternal peripheral blood and one sample from placental blood), from which 500µL of whole blood was stored in −20 °C freezers. Those frozen samples were used for RDT testing, consisting in one to four samples per woman depending on whether she completed the study follow-up until delivery.

Women with uncomplicated microscopic malaria were treated immediately with oral quinine in the 1st trimester and artemether-lumefantrine in the 2nd and 3rd trimesters. Those with severe malaria received intravenous artesunate until oral medication could be tolerated. Women received a long-lasting insecticide-treated net at their first ANC visit. Besides, the maternity staff was encouraged to administer at least three doses of IPTp as recommended by national guidelines [13].

### RDT and uRDT testing

The cRDT SD BIOLINE Malaria Ag Pf (05FK50, batch 05CDD019AA, Abbott USA) and the HRP2 ultrasensitive RDT Alere Malaria Ag Pf (05FK140, batch 05LDD001AA, Abbott USA) were used according to the manufacturer’s instructions. Each RDT reaction was performed in duplicate using 5 µL of previously frozen EDTA-anticoagulated venous whole blood and read in a blind manner. For each RDT reaction, the result of each test line was quantitatively recorded by two independent readers on a scale from 0 (no line) to 4 (strong line) using a standardized scoring chart. A *P. falciparum* positive blood sample was used as positive control to have a positive example of scales before RDTs testing. Therefore, a total of 4 readings were performed with each product for a given specimen. A specimen was considered RDT positive if at least one reader identified one repeat as positive (i.e. test line intensity > 0) and negative if all readings were negative (i.e. test line intensity = 0).

### Microscopy and qPCR testing

Microscopy and quantitative PCR (qPCR) testing was performed in the framework of RECIPAL study. Thick blood smears (TBS) were stained with Giemsa and parasitaemia was quantified by the Lambaréné method [14]. Blood smears were considered negative if no parasites were seen in all the 10µL TBS. The presence of *P. falciparum* was also tested in duplicate by a qPCR that targeted the 18S rDNA after 40 cycles of amplification [15, 16]. Starting from 50 µL of blood spotted on filter paper, the extraction procedure leads to a final volume of 150 µL extracted DNA. A test sampling of 5 µL extracted DNA thus corresponds to approximately 1.7 µL of blood. All parasite density estimates for this study were determined by qPCR. Parasitaemia was determined by extrapolation of cycle thresholds (Ct) from a standard curve generated with purified DNA from 3D7 *P. falciparum* infected erythrocyte culture. Samples without amplification (no cycle thresholds detected) were considered negative, and a density of 2 parasites/μL was assigned if amplification was observed out of the lower range of the standard curve (5 parasites/μL). Purified DNA 3D7 parasite strain was used as a positive control while negative control with no DNA template was run in all reactions. *Plasmodium falciparum* infections detected by qPCR, but not by microscopy, were classified as sub-microscopic. A quality check qPCR (using FTD Malaria, FastTrack Diagnostics) was performed in Hôpital Bichat (France) on same DNA extracts from a 10% randomly selected sub-sample. This reagent is for detection of *Plasmodium* spp. DNA. The limit of detection was estimated by the manufacturer of 0.1 target copy/µl of DNA extract. Considering that the PCR was carried out using 10 μL of DNA extract (equivalent to 3.4 μl of whole blood), the detection limit was equivalent to 0.3 parasites/μl of whole blood. If the sample displayed an exponential trace under cycle threshold 38, this sample was considered as positive.

### *Plasmodium falciparum* HRP2 quantification

HRP2 was quantified using a highly sensitive laboratory based quantitative bead suspension array (qSA) based on Luminex technology [17]. After incubating 2’000 magnetic beads per analyte with blood sample at 1:5 dilution, beads were sequentially incubated with in-house biotinylated antibody α-HRP2 (MBS832975, MyBiSource, San Diego, CL) and streptavidin-PE (42250-1ML, Sigma Aldrich, St. Louis, MO). After a final wash, a minimum of 50 microspheres per analyte were acquired using the Luminex xMAP^®^ 100/200 analyser (Luminex Corp., Austin, TX). After subtracting background (blank) values, median fluorescent intensities (MFI) were normalized to account for plate to plate variation and quantification was performed against a 5-parameter logistic (5-PL) regression curve consisting of recombinant protein HRP2 type A (890015, Microcoat GmbH, Germany).

### Statistical analysis

For both RDTs, Kappa coefficients were estimated to assess agreement among the different readings. The positivity rate by diagnostic test (qPCR, uRDT, cRDT and microscopy) as well as the proportion of sub-microscopic infections were calculated. The sensitivity, specificity, positive predictive value (PPV), negative predictive value (NPV), positive likelihood ratio (LR +) and negative likelihood ratio (LR-) of each RDT were assessed compared to qPCR considered as reference standard. Secondary analyses were performed to assess RDTs’ performance according to gravidity (primi- and secundigravidae *vs.* multigravidae), trimester of pregnancy (1st trimester, 3rd trimester, and delivery), type of blood sample (peripheral *vs.* placental blood at delivery), and symptoms (symptomatic *vs.* asymptomatic women). Symptomatic women were those who presented fever (axillary temperature ≥ 37.5 °C) or history of fever in the preceding 24 h, whether malaria infection was detected by qPCR, uRDT or cRDT. For each sub-group, sensitivity and specificity were compared between uRDT and cRDT using McNemar test for matched pairs. For all performance characteristics, exact binomial confidence intervals were computed. The geometric mean (95% Confidence Interval) of parasite density and HRP2 concentration were presented according to qPCR and RDT positivity.

The clinical impact of infections detected by the uRDT and not the cRDT was assessed. For that purpose, at each time-point of malaria screening using uRDT (i.e., at the 1st trimester, at the 3rd trimester and at delivery), women were categorized in four exclusive groups as detailed in Table 1. Groups 3 and 4 included both qPCR positive and qPCR negative specimens in order to assess the impact of uRDT and cRDT infections in a pragmatic way, regardless of qPCR result. The first analysis consisted in assessing the association between maternal haemoglogin (Hb) level and anaemia (defined as Hb level < 11 g/dL based on WHO recommendations for pregnant women) during pregnancy (in the 1st and 3rd trimester) with concomitant malarial infection according to women’s group. Mixed models were used in order to take into account the correlation between Hb concentrations measured in the same woman. The following variables were considered as potential confounding factors: maternal age (categorized according to the tertiles: < 23, 23–30,  > 30 years old), gravidity (primi-secundigravidae *vs.* multigravidae), pre-pregnancy body mass index (BMI), anaemia before conception (defined as Hb level < 12 g/dL based on WHO recommendations for non-pregnant women), maternal education level (illiterate *vs.* literate), socioeconomic level, and ethnicity (Toffin *vs.* other). Pre-pregnancy BMI was classified into low (< 18.5 kg/m^2^), normal (18.5−24.9 kg/m^2^) and high (> 25 kg/m^2^) according to WHO classification. Socioeconomic level was approximated using a synthetic score combining occupation and ownership of assets, which was then categorized according to the tertiles. All variables with a *P* value below 0.2 in univariate analysis were included in the multivariate analysis. Then, the variables were eliminated step-by-step using the backward selection procedure, leaving only those variables with a *P*-value < 0.05. Gravidity and trimester of pregnancy were forced in the final multivariate models. The second analysis consisted in assessing the association between birthweight and low birthweight (LBW, defined as < 2500 grams in live-birth babies) with malaria at delivery according to women’s group. Women were classified according to the highest group to which they belonged in either peripheral or placental blood. Because of convergence issues, probably due to the small sample size, both models were only adjusted for gravidity. Stata version 13 for Windows (Stata Corp., College Station, TX) was used for all statistical analyses.

**Table 1:** Women’s classification according to qPCR, uRDT and cRDT positivity

Group^a^	qPCR	uRDT	cRDT	Comment
1	Negative	Negative	Negative	Reference group
2	Positive	Negative	Negative	PCR positive infection
3	Positive or Negative	Positive	Negative	uRDT positive infection
4	Positive or Negative	Positive or Negative	Positive	cRDT positive infection

^a^At delivery, women were classified according to the highest group to which they belonged in either peripheral or placental blood; NB: specimens that were uRDT negative/cRDT positive (n = 2) were not classified in any of the groups

## Results

### Study participants characteristics

A total of 942 blood specimens were used for this study corresponding to 327 women who consented to participate and from whom sufficient archived whole blood samples were available. Primigravidae accounted for 7.7% of the study population (Table 2). Before pregnancy, 57.8% of women were anaemic (Hb level < 12 g/dL). During pregnancy, 66% of women received 2 or 3 doses of IPTp with SP. The proportion of women with at least one microscopic malaria infection during pregnancy was 40.0% (131/327), with 22.3% (71/319), 17.3% (52/300) and 14.3% (39/272) infected women in the 1st, 2nd and 3rd trimester of pregnancy, respectively. Thirty-eight percent and 58.5% of women were anaemic in the 1st and 3rd trimester of pregnancy, respectively (see Additional file 2). The prevalence of low birth weight was 8.0%. Women included in the present study had similar characteristics compared to the 411 women included in the RECIPAL study, except for malaria in pregnancy (defined as at least one microscopic infection), which was marginally higher in women included in the present study (40% *vs.* 34%, p = 0.10).

**Table 2:** General characteristics of the 327 pregnant women included in the uRDT study

General characteristics	Proportion or mean (SD)
Age (years)	
Mean (SD)	26.7 (5)
< 23	61 (18.8)
23–30	248 (76.3)
> 30	16 (4.9)
Ethnicity	
Toffin	73.1%
Aizo	13.6%
Other	13.3%
Education level	
Illiterate	69.7%
Socioeconomic level^a^	
Low (1st tertile)	111 (34.2)
Medium (2nd tertile)	134 (41.2)
High (3rd tertile)	80 (24.6)
Gravidity	
Primigravidae	7.7%
Secundigravidae	15.4%
Multigravidae	76.9%
HIV infection (n = 309)	1.7%
BMI (kg/m^2^)—WHO categories	
< 8, 5	9.3%
18, 5–24	66.7%
≥ 25	24.0%
Anaemia before conception^b^	57.8%
During pregnancy	
Number of IPTp intakes	
0	16%
1	19%
2	54%
3	12%
At least one microscopic malaria infection	
During pregnancy	40.0%
1st trimester	22.3% (71/319)
2nd trimester	17.3% (52/300)
3rd trimester	14.3% (39/272)

^a^Socioeconomic level was approximated using a synthetic score combining occupation and ownership of assets, which was then categorized according to the tertiles within the whole RECIPAL cohort

^b^Defined as a Hb level < 12 g/dL according to WHO recommendations for non-pregnant women

### Malaria infection rate by diagnostic test

**Fig. 1:**
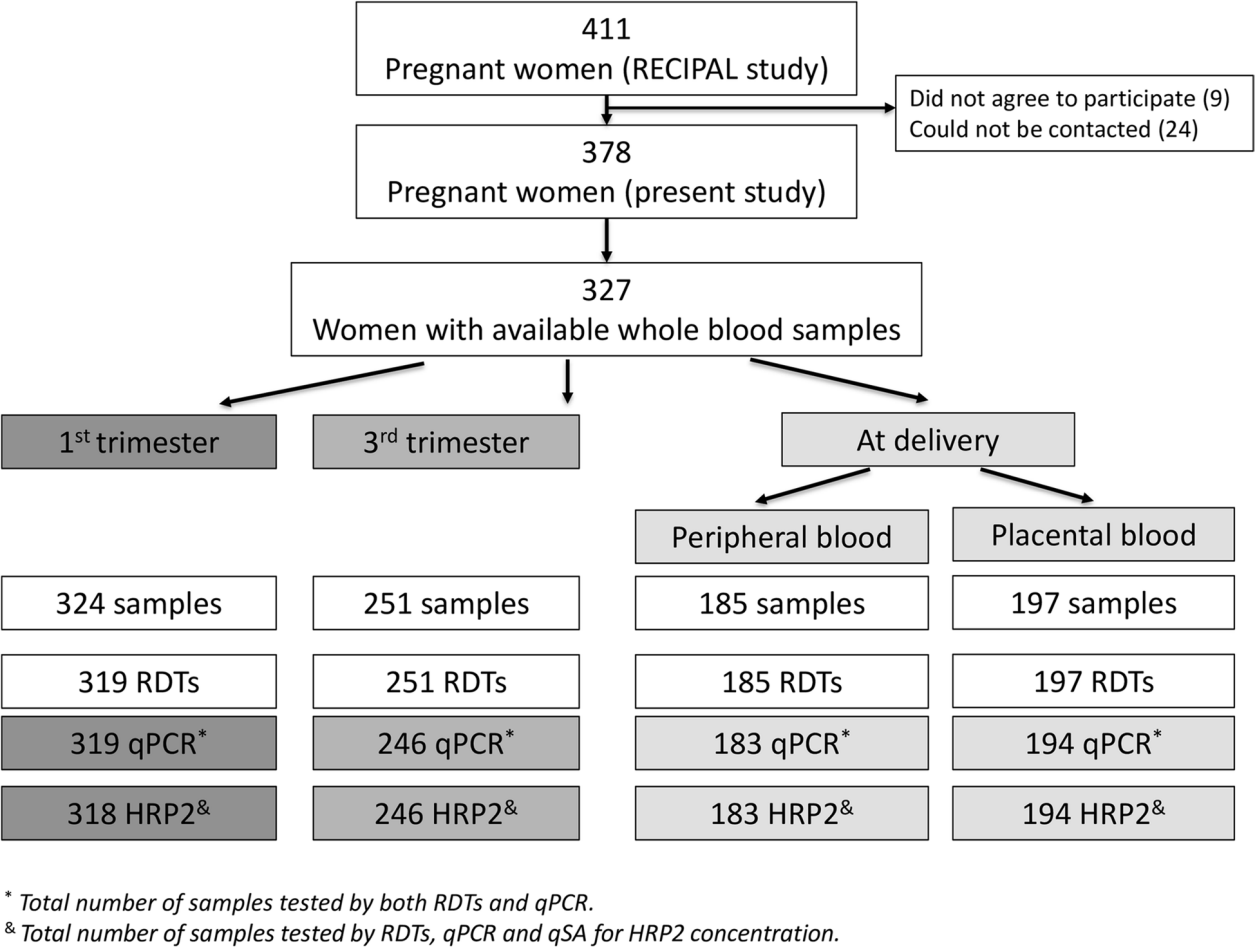
Flow diagram of included women and available specimens tested by uRDT, cRDT, qPCR and qSA for HRP2 concentration. RECIPAL study (Benin, 2014–2017)

A total of 319, 246, 183 and 194 specimens were tested with uRDT, cRDT and qPCR in the 1st trimester, in the 3rd trimester, at delivery in peripheral blood and in placental blood, respectively (Fig. 1). Mean (standard deviation [SD]) gestational age in the 1st and 3rd trimester was 11.7 (3) and 33 (3.2) weeks gestation, respectively. For uRDT and cRDT, kappa coefficients ranged from 96% to 99%; both RDTs were then considered as positive if at least one out of four readings was positive. Agreement between the initial qPCR and the quality check PCR was 92.6%. Discrepant results were probably due to a difference in the detection limit between both PCRs [18].

The overall positivity rate detected by uRDT (16.2%) was slightly lower than with qPCR (18.3%), but higher than with cRDT (11.6%) (Table 3). Irrespective of the diagnostic test, infection rates were consistently the highest in the 1st trimester of pregnancy. The overall proportion of sub-microscopic infection was 16.7%, with the highest prevalence in the first trimester of pregnancy (32%). Most infections detected during pregnancy or in peripheral blood at delivery (88.2%, 172/195) were asymptomatic.

**Table 3:** *Plasmodium falciparum* positivity rate by diagnostic test in blood samples collected during pregnancy and at delivery. RECIPAL study, 2014–2017

	Total (N specimens)	During pregnancy		At delivery	
		1st trimester^a^	3rd trimester^a^	Peripheral blood	Placental blood
Positivity rates in specimens tested with uRDT, cRDT and qPCR					
cRDT	11.6% (109/942)	15.7% (50/319)	10.6% (26/246)	8.2% (15/183)	9.3% (18/194)
uRDT	16.2% (153/942)	24.8% (79/319)	13.4% (33/246)	11.5% (21/183)	10.3% (20/194)
qPCR	18.3% (172/942)	33.5% (107/319)	15.0% (37/246)	9.8% (18/183)	5.2% (10/194)
Positivity rates in specimens tested with both microscopy and qPCR					
Microscopy	3.7% (34/926)	5.2% (16/310)	2.1% (5/244)	2.2% (4/180)	4.7% (9/192)
Sub-microscopic infection^b^	16.7% (149/992)	32.0% (94/294)	13.8% (33239)	8.0% (14/176)	4.4% (8/183)

^a^Mean (SD) gestational age (weeks of gestation): 11.7 (3) wg for specimens collected in the 1st trimester, 33 (3.2) wg for specimens collected in the 3rd trimester

^b^qPCR positive but thick blood smear negative specimens

### Performance of RDTs

**Fig. 2:**
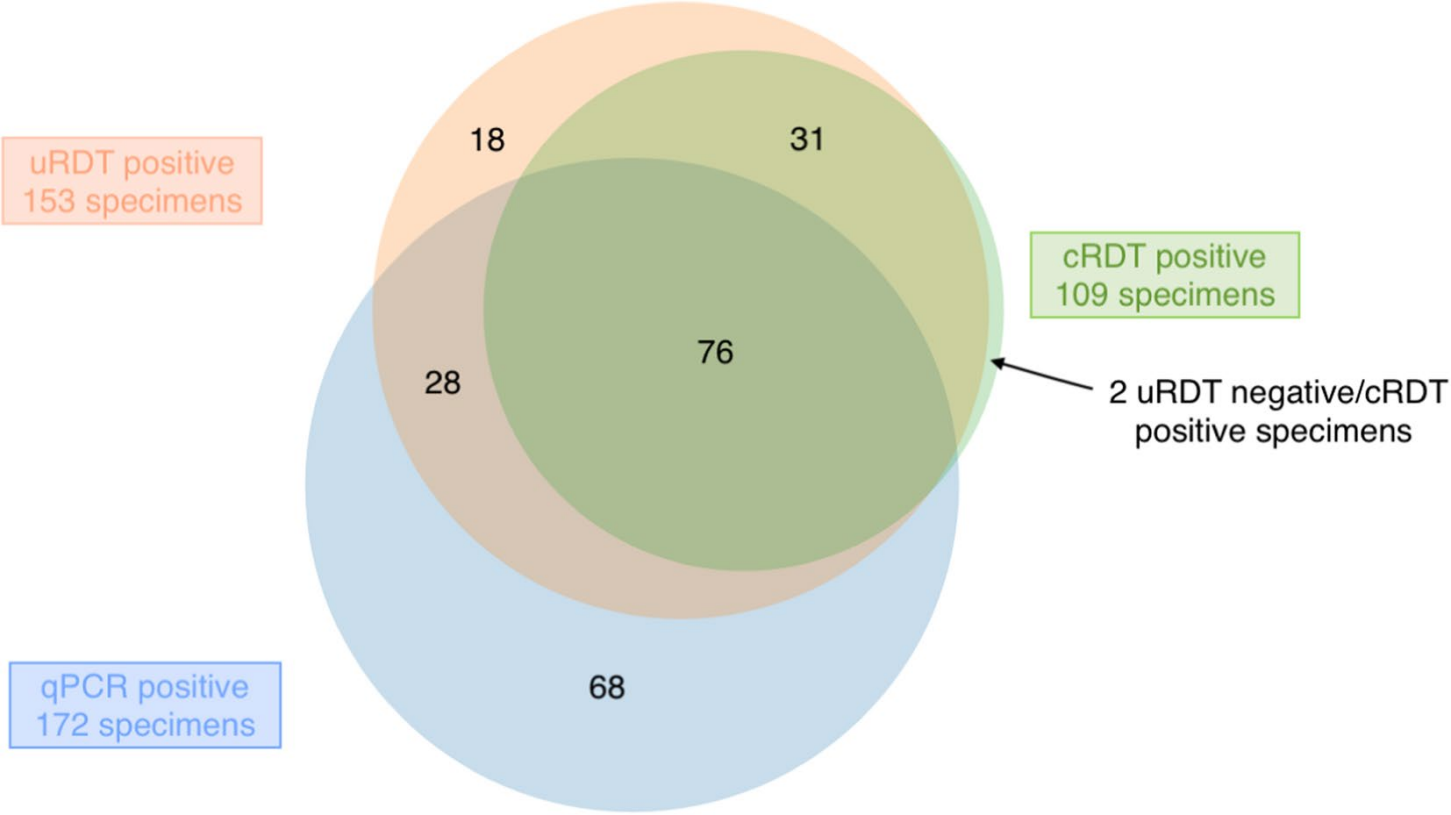
Venn diagram of *Plasmodium falciparum* positivity by uRDT, cRDT and qPCR. RECIPAL study (Benin, 2014–2017)

The uRDT identified all specimens—except two—with positive cRDT results, plus 46 additional specimens with negative cRDT results (30.0%, 46/153) (Fig. 2). Of these 46 specimens, 28 were also positive by qPCR; mean (95%CI) parasite density was 20.7 p/µL (10.8–39.6). Two specimens were cRDT positive and uRDT negative; both were negative by HRP2.

The uRDT sensitivity (60.5% (52.7−67.8)) was significantly higher than that of the cRDT (44.2% (36.6–51.9)) (Table 4). The difference in sensitivity between uRDT and cRDT was particularly high in the 1st trimester, in multigravidae and in asymptomatic women (Table 5). The uRDT specificity was marginally lower than the one of the cRDT (93.6% *vs.* 95.7%, respectively) (Table 4), with statistically significant differences for samples collected in the 1st trimester, in primi/secundigravidae, in multigravidae and in asymptomatic women (Table 5). The positive predictive value was slightly lower for uRDT as compared to cRDT (68.0% *vs.* 69.7%), but uRDT had a higher negative predictive value (91.4% *vs.* 88.5%). Finally, positive and negative likelihood ratios were similar between uRDT and cRDT (Table 4).

**Table 4:** Performance of uRDT and cRDT against qPCR. RECIPAL study, 2014–2017

	PCR			Value (95%CI)^a^					
	**(+)**	**(−)**	Total	Sensitivity^b^	Specificity^b^	PPV	NPV	Positive LR	Negative LR
cRDT									
**(+)**	76	33	109	44.2%	95.7%	69.7%	88.5%	10.3	0.6
**(−)**	96	737	833	(36.6–51.9)	(94.0–97.0)	(60.2–78.2)	(86.2–90.6)	(7.1–15)	(0.5–0.7)
uRDT									
**(+)**	104	49	153	60.5%	93.6%	68.0%	91.4%	9.5	0.4
**(−)**	68	721	789	(52.7–67.8)	(91.7–95.3)	(60.0–75.3)	(89.2–93.2)	(7.1–12.8)	(0.3–0.5)

*PPV* Predictive Positive Value, *NPV* Negative Predictive Value, *LR* Likelihood Ratio

^a^For all performance characteristics, exact binomial confidence intervals were computed

^b^P-value < 0.05 using McNemar test for matched pairs

**Table 5:** Performance of uRDT and cRDT against qPCR, according to the trimester of pregnancy, gravidity, and presence of fever

	URDT	CRDT	P-value^*a*^
Sensitivity (95% CI)			
1st trimester	57.0% (47.1–66.5)	38.3% (29.1–48.2)	< 10^−3^
3rd trimester	64.9% (47.5–79.8)	54.1% (36.9–70.5)	0.05
Delivery (peripheral blood)	83.3% (58.6–96.4)	61.1% (35.7–82.7)	0.05
Delivery (placental blood)	40.0% (12.2 –73.8)	40.0% (12.2–73.8)	–
Primi and Secundigravidae	56.5% (41.1–71.1)	43.5% (28.9–58.9)	0.01
Multigravidae (3 +)	63.6% (54.2–72.2)	44.9% (35.7–54.3)	< 10^−3^
Asymptomatic	60.8% (52.3–68.9)	43.4% (35.1–51.9)	< 10^−3^
Symptomatic^*^	66.7% (41.0 –86.7)	50.0% (26.0–74.0)	0.08
Specificity (95% CI)			
1st trimester	91.5% (86.9–94.9)	95.7% (92.1 –98.0)	0.003
3rd trimester	95.7% (92.0–98.0)	97.1% (93.8–99.0)	0.08
Delivery (peripheral blood)	96.4% (92.2–98.7)	97.6%(94.0–99.3)	0.16
Delivery (placental blood)	91.3% (86.3–94.9)	92.4% (87.6–95.8)	0.41
Primi and Secundigravidae	93.9% (88.4–97.3)	97.0% (92.4–99.2)	0.05
Multigravidae (3 +)	94.5% (92.1–96.3)	96.3% (94.3–97.8)	0.007
Asymptomatic	94.6% (92.3–96.4)	97.1% (95.3–98.4)	< 10^−3^
Symptomatic^b^	92.2% (82.7–97.4)	93.7% (84.8–98.3)	0.32

RECIPAL study, 2014–017

^a^Comparison of uRDT and cRDT performance using McNemar test for matched pairs. Exact binomial confidence intervals were computed

^b^Presence of fever (axillary temperature ≥ 37.5 °C) or history of fever in the preceding 24 h, whether or not infected with malaria

### Parasite density and HRP2 concentration

**Fig. 3:**
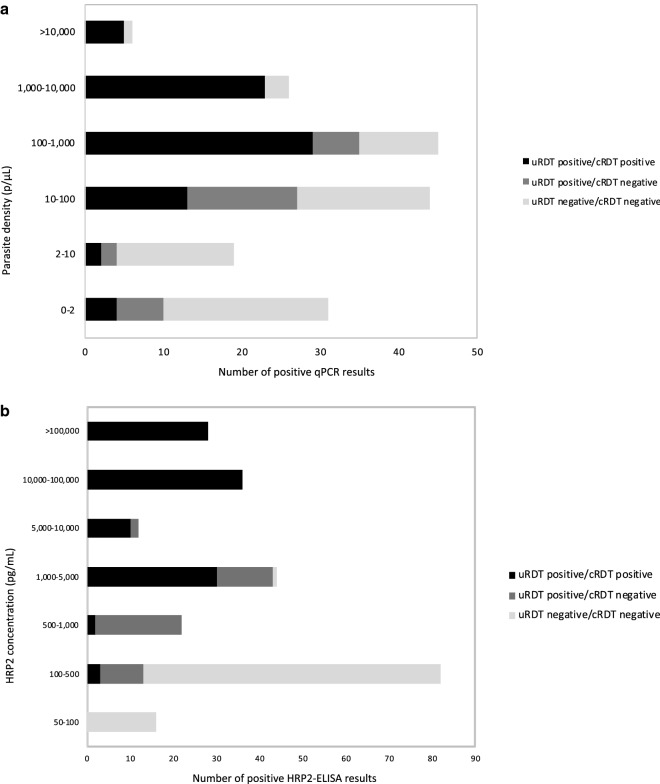
Distribution of specimens by parasite density (**a**) and HRP2 concentration (**b**), according to Das et al. [19]. **a** The outer clear bars represent the specimens that were positive by qPCR only; the gray bars are the number of specimens positive by uRDT and the black bars are the number of specimens positive by cRDT. **b** The outer clear bars represent the specimens that were positive by quantitative HRP2 assay, but not by RDTs; the gray bars are the number of specimens positive by uRDT and the black bars are the number of specimens positive by cRDT

Among the 941 specimens with HRP2 data as well as concomitant RDTs and qPCR results, the geometric mean (95%CI) HRP2 concentration in positive specimens (238/941) was 2139 (1486–3079) pg/mL. Forty specimens were qPCR positive but negative for HRP2 (Table 6); all of them tested negative by both RDTs and had low parasite densities (geometric mean (95%CI): 18.6 p/µL (7.3–47.5)). A total of 106 specimens were qPCR negative but positive for HRP2; the concentration of HRP2 was higher when both RDTs were positive. Among the HRP2 positive samples, the proportion of HRP2 positive/qPCR negative samples was higher in the 3rd trimester (44% (22/50)) and at delivery (58% (22/38) in maternal peripheral blood and 85% (35/41) in placental blood) than in the first trimester (24% (27/111)). Among the 5 women at delivery who were qPCR negative in peripheral blood, but positive by uRDT and had detectable levels of HRP2 (in peripheral blood), all of them were negative by qPCR in placental blood. Figure 3 presents the distribution of specimens, and their positivity regarding uRDT and cRDT, according to parasite density and HRP2 concentration. The proportion of infections that were detected by uRDT but not by cRDT increased with decreases in HRP2 levels. However, at very low concentrations of the antigen, both the uRDT and cRDT tended to be negative. The same trend was observed with decreasing parasite densities.

**Table 6:** HRP2 concentration and parasite density according to qPCR, uRDT and cRDT positivity. RECIPAL study, 2014–2017

	qPCR positive						qPCR negative			
	N	HRP2 positive	Mean HRP2 (pg/mL)^b^	95%CI	Mean qPCR density (parasites/µL) ^b^	95%CI	N	HRP2 positive	Mean HRP2 (pg/mL)^b^	95%CI
cRDT and uRDT positive	76	76	27,911	16,791–46,395	532	324–874	31	31^c^	16,881	5597 –50,914
cRDT negative and uRDT positive	28	28	1049	757–1,455	20.7	10.8 –39.6	18	17^c^	616	427–890
cRDT and uRDT negative	68	28	191	157–233	17	9 –30	718	58	160	135– 189

^a^Out of 721 qPCR-negative specimens, 3 were not included in the Table 2 of them were cRDT-positive and uRDT-negative; and one (RDTs-negative) was not tested for HRP2

^b^Geometric mean (95% confidence interval); HRP2 concentration and parasite density by qPCR calculated in positive HRP2 (qPCR) specimens

^c^Among the women at delivery who were qPCR negative in peripheral blood, but positive by uRDT and had detectable levels of HRP2, all of them (5/5) were negative by qPCR in placental blood

### Clinical impact of uRDT-detected malaria infections

Poor maternal and birth outcomes were more likely in women with uRDT-detected infections (see Additional file 2). In multivariate analysis, women with uRDT infections had a 3.4-times increased risk of anaemia during pregnancy than uninfected women (aOR (95%CI) = 3.44 (1.29–9.19), p = 0.01) (Table 7). This excess risk was higher than the one associated with cRDT infections (aOR (95%CI) = 2.03 (1.01–4.01), p = 0.05), but the difference was not statistically significant (p = 0.64). There was no association between qPCR positive and RDT negative infections and maternal anaemia. Similar results were found with maternal Hb level considered as a continuous variable (see Additional file 3).

**Table 7:** Association between malaria and maternal and child outcomes (maternal anaemia during pregnancy and low birthweight)

	Maternal anaemia^a^			Low birthweight^b^		
	AOR	95%CI	P value	AOR	95%CI	P value
Malaria^c^						
Group 1–No infection	Ref			Ref		
Group 2–qPCR infection	1.59	0.76–3.45	0.24	1	NA	
Group 3–uRDT infection	3.44	1.29–9.19	0.01	5.27	0.46; 57.14	0.17
Group 4–cRDT infection	2.03	1.01-4.01	0.05	2.29	0.57; 9.17	0.24
Gravidity						
Primi-secundigravidae	Ref			Ref		
Multigravidae	0.64	0.34–1.21	0.17	0.54	0.15; 1.88	0.33
Trimester of pregnancy						
1st trimester	Ref					
3rd trimester	3.76	2.30–6.13	< 10^−3^			
Ethnicity^d^	2.78	1.48–5.23	0.002	–	–	–

RECIPAL, 2014–2017

*NA* not applicable

^a^Maternal anaemia (Hb level < 11 g/dL) in the 1st or 3rd trimester of pregnancy. Final logistic mixed regression model adjusted for gravidity, ethnicity and trimester of pregnancy (n = 559 observations, 319 women); aOR: adjusted Odd’s ratio

^b^Low birthweight (< 2500 grams), twins and stillbirths excluded. Final logistic regression model adjusted for gravidity (n = 175)

^c^Malaria status (1) at the time of Hb level determination in the 1st and 3rd trimesters of pregnancy or (2) at delivery based on diagnostic tests positivity in maternal peripheral and placental blood. Women were classified according to the highest group to which they belonged in either peripheral or placental blood (e.g. women classified in group 3 based on peripheral blood and in group 4 based on placental blood were at the end classified in group (4); Groups 1 to 4 defined as described in Table 1

^d^Ethnicity: Toffin (considered as the reference) vs. others

Women with uRDT infection at delivery had children with a higher risk of LBW (adjusted OR (95%CI) = 5.27 (0.46–57.14), p = 0.17) compared with uninfected women, but this association did not reach statistical significance (Table 7). A similar result was found when birthweight was considered as a continuous variable (see Additional file 3).

## Discussion

For the first time, the performance of a malaria RDT with a significantly improved analytical sensitivity as compared to commonly used RDTs [19–21] was evaluated in pregnant women living in a high malaria transmission area. Using qPCR as the reference standard, this uRDT had a significantly better sensitivity (60.5% (52.7−67.8)) than the studied cRDT (44.2% (36.6−51.9)). uRDT specificity was slightly lower (93.6% (91.7–95.3)) than for cRDT (95.7% (94.0–97.0)). The gain in sensitivity was particularly high and statistically significant in the 1st trimester of pregnancy, in multigravidae and in asymptomatic women (range, 29%–33%). In addition, our results indicate that infections which can be detected by uRDT but not by cRDT, were associated with maternal anaemia, suggesting that the use of more sensitive malaria RDT in the context of pregnancy might provide clinical benefits for the pregnant women, even in a context where IPTp-SP is made available.

Overall, 18.3% of specimens were qPCR positive, compared to 16.2% and 11.6% with uRDT and cRDT, respectively. The highest positivity rates were found for samples collected in the first trimester of pregnancy, before IPTp administration. In contrast, persistence of HRP2 (with qPCR negative) was observed in the 3rd trimester probably due to the clearance of parasites by IPTp. At delivery, positivity rate by qPCR was particularly low in placental blood compared to peripheral blood, possibly due to placental inhibitors or a high concentration of chelex in placental blood. Also, it was low compared with positivity rate by RDT. It is plausible that all infections detected in the peripheral blood were not necessarily of the placental type, and the RDTs did not make the difference by covering a wider parasite biomass.

The uRDT sensitivity ranged between 40.0% and 83.3% depending on the trimester of pregnancy, presence of symptoms and gravidity. The highest sensitivity rates were found for peripheral specimens collected at delivery (83.3%) and in symptomatic women (66.7%), but confidence intervals were large due to small sample sizes and low numbers of positive specimens in these two groups. The overall uRDT sensitivity in our study (60.5% (52.7–67.8)) was lower than the one reported by Vasquez et al. in Colombian pregnant women (85.7% (70.6–93.7)) [11]. The gain in performance between the cRDT and the uRDT is expected to vary from settings to settings, depending on the proportion of the infection that fall within the added detection window of the more sensitive uRDT [19, 22]. In this study, the relative gain in sensitivity was about 1.3 fold, similarly to previously reported data in the study by Vasquez et al. [11]. This improvement was particularly evident for samples collected in the first trimester of pregnancy. Such a high gain in sensitivity probably reflected the high proportion of sub-microscopic infections at this period.

Only 28 (41%) out of the 68 samples which were positive by qPCR but negative by uRDT had detectable levels of HRP2. However, the HRP2 concentrations were very low, explaining the negative uRDT result. Compared to samples which were negative both by cRDT and uRDT, higher HRP2 levels were detected in samples which were positive by uRDT but not by cRDT (5.5-fold increase), and positive by both RDTs (146-fold increase). HRP2 was detected in 48 of the 49 samples that were positive by uRDT but negative by qPCR. The discordance in the 40 samples which were HRP2 negative and qPCR positive could be due to the presence of gametocytes or *pfhrp2* deletions, which could not be investigated [23]. The presence of HRP2 in qPCR negative samples could be explained either by infections below the qPCR detection limit or by the persistence of the antigen from a recently cleared infection [23]. Indeed, as suggested by the observation that all (n = 5) the women at delivery who were qPCR negative in peripheral blood but positive by uRDT and had detectable levels of HRP2, were also negative by qPCR in placental blood. In regards to qPCR detection limit, uRDT and cRDT sensitivity might have been lower by using an ultrasensitive PCR as gold standard, with probably a higher gain in sensitivity for uRDT compared to cRDT.

Women infected with malaria detected only by uRDT and not by cRDT, were at higher risk of anaemia compared to uninfected women, highlighting the clinical relevance of detecting low density malaria infections in pregnancy. The systematic treatment of microscopic infections during RECIPAL study may have reduced the overall effect of cRDT infections on Hb level. Also, the analysis suggested that uRDT infections at delivery were associated with a five times higher risk of LBW, but this association was not statistically significant. Because of the small numbers of LBW babies and women infected with malaria at delivery, the analysis was probably underpowered to detect any significant association. In any case, this result has to be taken with caution for the two following reasons. First, malaria at delivery was the main exposure, which may not reflect malaria history during pregnancy because of a high IPTp coverage and the systematic treatment of microscopic infections in RECIPAL study. Second, one cannot exclude that women with a positive uRDT infection at delivery had a cRDT positive infection during pregnancy.

## Conclusion

In conclusion, these results are complementary to those previously reported in a low malaria transmission area. They confirm the higher overall performance of uRDT, compared to the studied conventional RDT, to detect *P. falciparum* infections in pregnant women. Indeed, the gain in sensitivity associated with uRDT exceeded its loss in specificity, with undetected infections possibly associated with poor pregnancy outcomes. The higher detection of infections using uRDT compared to cRDT was particularly evident in the first trimester of pregnancy, when sub-microscopic infections are the most prevalent. Since IPTp-SP is recommended from the 2nd trimester onwards, using uRDT for identifying those women who are infected with malaria in the first trimester of pregnancy may be especially relevant. Although uRDT will still miss almost 50% of the infected women at this period, women detected as infected with malaria using uRDT may be the ones at risk of poor outcomes. Overall, these encouraging results call for further prospective studies assessing uRDT performance and clinical relevance under field conditions.

## Supplementary information

**Additional file 1**. Type and volume of samples that were used for uRDT and cRDT testing, HRP2-assay and qPCR. RECIPAL, 2014–2017.

**Additional file 2**. Maternal and birth outcomes, according to type of concomitant malaria infection. RECIPAL, 2014–2017.

**Additional file 3**. Association between malaria and maternal and child outcomes (maternal Hb level during pregnancy and birthweight). RECIPAL, 2014–2017.

## Data Availability

The dataset generated and analysed during the current study will be available in Open Science Framework repository only after acceptance of the manuscript for publication.
