# Correction to: Prevalence and clinical impact of malaria infections detected with a highly sensitive HRP2 rapid diagnostic test in Beninese pregnant women

**DOI:** 10.1186/s12936-020-03400-8

**Published:** 2020-09-07

**Authors:** Valérie Briand, Gilles Cottrell, Nicaise Tuikue Ndam, Xavier Martiáñez–Vendrell, Bertin Vianou, Atika Mama, Bienvenue Kouwaye, Sandrine Houzé, Justine Bailly, Erasme Gbaguidi, Darius Sossou, Achille Massougbodji, Manfred Accrombessi, Alfredo Mayor, Xavier C. Ding, Nadine Fievet

**Affiliations:** 1 Institut de Recherche Pour le Développement (IRD), University of Bordeaux, Inserm, UMR 1219, 146 Rue Léo-Saignat, 33076 Bordeaux Cedex, France; 2 Université de Paris, MERIT, IRD, 75006 Paris, France; 3 ISGlobal, Hospital Clínic of Barcelona, Universitat de Barcelona, Barcelona, 08036 Spain; 4 Institut de Recherche Clinique du Bénin (IRCB), Cotonou, Benin; 5 AP-HP, Centre National de Référence sur le paludisme, Hôpital Bichat-Claude-Bernard, 75017 Paris, France; 6 Faculty of Infectious and Tropical Diseases, Disease Control Department, London School of Hygiene and Tropical Medicine, WC1E 7HT London, UK; 7 FIND, 1202 Geneva, Switzerland

## **Correction to:** Malar J (2020) 19:18810.1186/s12936-020-03261-1

Following publication of the original article [1], the authors flagged that there is an error present in Table 5.

As a result of an error in the statistical program used to categorize women based on ‘gravidity’, some ‘secundigravidae’ have been (erroneously) classified as ‘multigravidae’.

Consequently, the performance values based on gravidity, which are secondary results, are incorrect in the table.

To amend this error, the corrected Table 5 is provided in this correction.

**Table 5:** Performance of uRDT and cRDT against qPCR, according to the trimester of pregnancy, gravidity, and presence of fever

	uRDT	cRDT	P value^a^
Sensitivity (95% CI)			
1st trimester	57.0% (47.1–66.5)	38.3% (29.1–48.2)	<10^−3^
3rd trimester	64.9% (47.5–79.8)	54.1% (36.9–70.5)	0.05
Delivery (peripheral blood)	83.3% (58.6–96.4)	61.1% (35.7–82.7)	0.05
Delivery (placental blood)	40.0% (12.2–73.8)	40.0% (12.2–73.8)	–
Primi and Secundigravidae	57.4% (42.2–71.7)	44.7% (30.2–59.9)	0.01
Multigravidae (3+)	61.6% (52.5–70.2)	44.0% (35.1–53.2)	<10^−3^
Asymptomatic	60.8% (52.3–68.9)	43.4% (35.1–51.9)	<10^−3^
Symptomatic*	66.7% (41.0–86.7)	50.0% (26.0–74.0)	0.08
Specificity (95% CI)			
1st trimester	91.5% (86.9–94.9)	95.7% (92.1–98.0)	0.003
3rd trimester	95.7% (92.0–98.0)	97.1% (93.8–99.0)	0.08
Delivery (peripheral blood)	96.4% (92.2–98.7)	97.6% (94.0–99.3)	0.16
Delivery (placental blood)	91.3% (86.3–94.9)	92.4% (87.6–95.8)	0.41
Primi and Secundigravidae	93.5% (88.5–96.9)	96.1% (91.8–98.6)	0.04
Multigravidae (3+)	93.7% (91.4–95.5)	95.6% (93.7–97.1)	0.003
Asymptomatic	94.6% (92.3–96.4)	97.1% (95.3–98.4)	<10^−3^
Symptomatic^b^	92.2% (82.7–97.4)	93.7% (84.8–98.3)	0.32

RECIPAL study, 2014–2017

^a^ Comparison of uRDT and cRDT performance using McNemar test for matched pairs. Exact binomial confidence intervals were computed

^b^ Presence of fever (axillary temperature ≥ 37.5 °C) or history of fever in the preceding 24 h, whether or not infected with malaria

Finally, in addition to the above, the author name Nicaise Tuikue Ndam has been misspelled in the article as ‘Nicaise Tuike Ndam’; please find the corrected name in this correction.

The authors apologize for any inconvenience caused.
