# Supplementary material for: Prevalence and clinical impact of malaria infections detected with a highly sensitive HRP2 rapid diagnostic test in Beninese pregnant women
Source: Malar J. 2020 May 24;19:188. doi: 10.1186/s12936-020-03261-1 (PMC7247134; doi:10.1186/s12936-020-03261-1)
Supplement: Supplementary file 1 — Additional file 1. Type and volume of samples that were used for uRDT and cRDT testing, HRP2-assay and qPCR. RECIPAL, 2014–2017. [file 12936_2020_3261_MOESM1_ESM.docx]

**Additional file 1.** Type and volume of samples used for uRDT, cRDT and qPCR testing, thick blood smear (TBS) and HRP2-assay.

|  | Type of blood sample | Volume drawn | Timing during pregnancy | Storage | Volume used for testing |
| --- | --- | --- | --- | --- | --- |
| TBS | Capillary | 10µL whole blood | Monthly | None |  |
| qPCR | Capillary or Venous | 50µL whole blood on DBS | Monthly | -20°C | 5µL extracted DNA |
| uRDT | Venous / Placental | 500µL whole blood in EDTA tube | Three time-points : 1^st^ and 3^rd^ trimesters, and at delivery (peripheral and placental blood) | -20°C | 5µL/reaction |
| cRDT | Venous / Placental | 500µL whole blood in EDTA tube | Three time-points : 1^st^ and 3^rd^ trimesters, and at delivery (peripheral and placental blood) | -20°C | 5µL/reaction |
| HRP2 | Venous | 500µL whole blood in EDTA tube | Three time-points : 1^st^ and 3^rd^ trimesters, and at delivery (peripheral and placental blood) | -80°C | 35µL/reaction |

*TBS: Thick blood smear; DBS : Dried blood spot*
