# Supplementary material for: Prevalence and clinical impact of malaria infections detected with a highly sensitive HRP2 rapid diagnostic test in Beninese pregnant women
Source: Malar J. 2020 May 24;19:188. doi: 10.1186/s12936-020-03261-1 (PMC7247134; doi:10.1186/s12936-020-03261-1)
Supplement: Supplementary file 2 — Additional file 2. Maternal and birth outcomes, according to type of concomitant malaria infection. RECIPAL, 2014–2017. [file 12936_2020_3261_MOESM2_ESM.docx]

**Additional file 2**. Maternal and birth outcomes, according to type of concomitant malaria infection.

|  | Maternal anaemia | | Maternal Hb level | | Birthweight^b^ | LBW^b^ |
| --- | --- | --- | --- | --- | --- | --- |
|  | In the 1^st^ trimester^a^  % (n) | In the 3^rd^ trimester^a^  % (n) | In the 1^st^ trimester^a^  Mean (CI95%) | In the 3^rd^ trimester^a^  Mean (CI95%) | Mean (CI95%) | % (n) |
| Malaria^c^ |  |  |  |  |  |  |
| Group 1 – No infection | 31.6 (61/193) | 55.9 (109/195) | 11.6 (11.4-11.8) | 10.9 (10.8-11.1) | 3030 (2972-3088) | 6.9 (10/145) |
| Group 2 – qPCR infection | 50.0 (23/46) | 46.1 (6/13) | 11.2 (10.9-11.6) | 11.1 (10.7-11.5) | 3050 (2702-3399) | 0.0 (0/5) |
| Group 3 – uRDT infection | 55.2 (16/29) | 85.7 (6/7) | 10.9 (10.5-11.4) | 10.4 (90.8-11.0) | 2835 (2148-3523) | 25.0 (1/4) |
| Group 4 – cRDT infection | 44.0 (22/50) | 76.9 (20/26) | 11.1 (10.8-11.4) | 10.5 (10.2-10.8) | 3027 (2766-3288) | 14.3 (3/21) |
| Overall | 38.4 (122/318) | 58.5 (141/241) | 11.4 (11.3-11.5) | 11.8 (9.3-14.2) | 3026 (2968-3083) | 8.0 (14/175) |

**^a^** *Maternal anaemia: Hb level < 11g/dL (as recommended by the WHO for pregnant women) ; Hb level determined in the 1^st^ or 3^rd^ trimester of pregnancy.*

**^b^** *LBW: low birthweight (<2,500 grams); twins and stillbirths excluded.*

^c^ *Malaria status at the time of 1) Hb level (maternal anaemia) determination in the 1^st^ and 3^rd^ trimesters of pregnancy, and 2) at delivery in peripheral and placental blood for birth outcomes (LBW and birthweight); Groups 1 to 4 defined as described in Table 1.*
