# Supplementary material for: Prevalence and clinical impact of malaria infections detected with a highly sensitive HRP2 rapid diagnostic test in Beninese pregnant women
Source: Malar J. 2020 May 24;19:188. doi: 10.1186/s12936-020-03261-1 (PMC7247134; doi:10.1186/s12936-020-03261-1)
Supplement: Supplementary file 3 — Additional file 3. Association between malaria and maternal and child outcomes (maternal Hb level during pregnancy and birthweight). RECIPAL, 2014–2017. [file 12936_2020_3261_MOESM3_ESM.docx]

**Additional file 3**. Association between malaria and maternal and child outcomes (maternal Hb level during pregnancy and birthweight). RECIPAL, 2014-2017.

|  | Maternal Hb level^a^ | | | Birthweight^b^ | | |
| --- | --- | --- | --- | --- | --- | --- |
|  | aCoeff | 95%CI | Pvalue | aCoeff | 95%CI | Pvalue |
| **Malaria**^c^ |  |  |  |  |  |  |
| Group 1 – No infection | Ref |  |  | Ref |  |  |
| Group 2 – qPCR infection | -0.16 | -0.46 ; 0.14 | 0.29 | 19.9 | -319.9 ; 359.7 | 0.91 |
| Group 3 – uRDT infection | -0.53 | -0.90 ; -0.16 | 0.005 | -209.8 | -589.6 ; 169.9 | 0.28 |
| Group 4 – cRDT infection | -0.43 | -0.69 ; -0.16 | 0.002 | -3.9 | -178.3 ; 170.5 | 0.97 |
| **Gravidity** |  |  |  |  |  |  |
| Primi-secundigravidae | Ref |  |  | Ref |  |  |
| Multigravidae | 0.12 | -0.14 ; 0.37 | 0.37 | 74.0 | -67.6 ; 215.6 | 0.31 |
| **Timing during pregnancy** |  |  |  | - | - | - |
| 1^st^  trimester | Ref |  |  |  |  |  |
| 3^rd^ trimester | -0.60 | -0.76 ; -0.44 | <10^-3^ |  |  |  |
| **Ethnicity**^d^ | -0.43 | -0.67 ; -0.19 | <10^-3^ | - | - | - |
| Constant | 11.77 | 11.47; 12.07 |  | 2971 | 2843; 3099 |  |

^a^ *Hb level (g/dL) determined in the 1^st^ or 3^rd^ trimester. Linear mixed regression model adjusted for gravidity, ethnicity and timing during pregnancy (n=559 observations, 319 women); aCoeff: Adjusted Coefficient.*

^b^ *Birthweight, twins and stillbirths excluded. Linear regression model adjusted for gravidity (n=170).*

^c^ *Malaria status 1) at the time of Hb level determination in the 1^st^ and 3^rd^ trimesters of pregnancy or 2) at delivery based on diagnostic tests positivity in maternal peripheral and placental blood ; Groups 1 to 4 defined as described in Table 1.*

^d^ *Ethnicity: Toffin (considered as the reference) vs. others*
